# New species and new records of bryozoan species from fouling communities in the Madeira Archipelago (NE Atlantic)

**DOI:** 10.1007/s12526-023-01355-y

**Published:** 2023-07-07

**Authors:** Javier Souto, Patrício Ramalhosa, Jasmine Ferrario, Lydia Png-Gonzalez, Soledad Álvarez, Ignacio Gestoso, Natacha Nogueira, João Canning-Clode

**Affiliations:** 1 Institut Für Paläontologie, Geozentrum, Universität Wien, Josef-Holaubek-Platz 2, 1090 Vienna, Austria; 2 MARE (Marine and Environmental Sciences Centre)/ARNET (Aquatic Research Network), Agência Regional Para o Desenvolvimento da Investigação Tecnologia E Inovação (ARDITI) Funchal, Madeira, Portugal; 3 Department of Earth and Environmental Sciences, University of Pavia, Via S. Epifanio 14, 27100 Pavia, Italy; 4 Centro Oceanográfico de Baleares (IEO, CSIC), Muelle de Poniente S/N, 07015 Palma, Spain; 5 Department of Biology, Faculty of Marine and Environmental Sciences of University of Cádiz, Puerto Real, Spain; 6 Smithsonian Environmental Research Center, 647 Contees Wharf Road, Edgewater, MD 21037 USA; 7 Interdisciplinary Centre of Marine and Environmental Research, Av. General Norton de Matos, 4450-238 Matosinhos, Portugal; 8 Regional Directorate for the Sea, Lota Do Funchal 1º Piso, Rua Virgílio Teixeira, 9004-562 Funchal, Madeira Portugal

**Keywords:** Artificial substrate, Biofouling, First records, New species, *Amathia*, *Crisia*

## Abstract

Hull fouling is considered to be the most significant vector of introduction of marine non-indigenous species (NIS) in the Madeira Archipelago (NE Atlantic) because these islands provide a vital passage route for many ships. The transfer of species between boat hulls and artificial substrates in marinas is known to be high. Bryozoans are among the most common groups of marine invertebrates growing on this type of substrate. In recent years, significant advances have been made in our knowledge about the biodiversity of bryozoans in the Madeira Archipelago. Nonetheless, the currently recognized numbers remain far from reflecting the actual bryozoan species richness. In this context, we examine bryozoan samples stemming from NIS monitoring surveys on artificial substrates along the southern coast of the Madeira Archipelago, in four recreational marinas and in two offshore aquaculture farms. This has yielded new information about ten bryozoan species. Two of them,* Crisia noronhai* sp. nov. and *Amathia maderensis* sp. nov., are described for the first time, although at least the first one was previously recorded from Madeira but misidentified. *Bugula ingens*, *Cradoscrupocellaria insularis*, *Scruparia ambigua*, and *Celleporaria brunnea* are recorded for the first time in Madeira. Moreover, the material of *C. brunnea* was compared with the type, and a biometric analysis was performed with material from the Atlantic and Mediterranean. All samples identified as *C. brunnea* in both regions are the same species, and the variations described in the literature apparently reflect high intracolonial variability. Finally, we provide new information for the descriptions of 4 additional bryozoans, namely, *Crisia* sp. aff*. elongata*, *Cradoscrupocellaria bertholletii*, *Scrupocaberea maderensis*, and *Tricellaria inopinata*.

## Introduction

The Madeira Archipelago is a group of Portuguese volcanic islands located in the NE Atlantic Ocean, 700 km off the Moroccan coast. Madeira is the largest of the two inhabited islands, with 144 km of coastline, whereas Porto Santo is located about 42 km northeast of Madeira island with about 33 km of coastline (Ramalhosa et al. 2019). Historically, the archipelago has provided a vital passage route for many ships between Europe, America and Africa because of its unique geographical position in the Atlantic Ocean, which offers an important port for re-fueling and rest stops (Castro et al. 2020).

Pioneer works on the bryozoan fauna from the Madeira Archipelago date back to the late nineteenth century (Busk 1858a, 1858b, 1859, 1860, 1861; Hincks 1880; Johnson 1897; Waters 1899; Norman 1909). Since then, several species have been added to this list, with most of the new records detected during the last two decades (d’Hondt 1985, Alves and Cocito 2002, Berning and Kuklinski 2008, Berning et al. 2008, Wirtz and Canning-Clode 2009, Berning 2012, Souto et al. 2014). In fact, Berning (2012) listed 140 species of bryozoans in Madeira and considered the island a “hotspot” of bryozoan diversity compared to other nearby regions. Furthermore, the author indicated that the known bryozoan species list for the archipelago was likely an underestimate (Berning 2012).

Accordingly, our knowledge of cheilostome bryozoan species from the Madeira Archipelago is far from complete, as several new records have been detected and inventoried in recent years due to comprehensive nonindigenous species (NIS) monitoring surveys, particularly in marinas along the southern coast (Canning-Clode et al. 2013a; Ramalhosa et al. 2017a, 2019). These recent monitoring surveys have increased sampling efforts and resulted in the discovery and description of three bryozoan species new to science (Souto et al. 2015, 2018). Nevertheless, the lack of a comprehensive checklist here makes the detection and dating of new anthropogenic introductions a complex challenge. Today, however, an estimated 150 species have been recorded. The increased detection in recent years also benefited from using Scanning Electron Microscopy (SEM) for identification instead of the optical microscopy used in past studies (Berning 2012; Souto et al. 2014; Ramalhosa et al. 2017a).

International shipping is one of the most significant vectors contributing to the spread and establishment of marine NIS, thus representing a significant threat to biodiversity in coastal marine ecosystems worldwide (Molnar et al. 2008; Carlton and Ruiz 2016). Furthermore, biological invasions in the sea have been increasing in recent years because maritime traffic facilitates the transport and arrival of NIS into new regions through the ballast water on ships and/or hull fouling (Kaluza et al. 2010, Clarke Murray et al. 2014). Indeed, hull fouling is the most significant vector of the introduction of marine NIS in the Madeira Archipelago coastal waters (Canning-Clode et al. 2013a; Ramalhosa et al. 2014, 2017a, 2017b, 2019; Ramalhosa and Canning-Clode 2015; Souto et al. 2018). Moreover, offshore aquaculture activities facilitate the local dispersion of NIS (Nunes et al. 2014; Campbell et al. 2017), posing a serious environmental threat to biodiversity and ecosystem function (Mack et al. 2000; Png-Gonzalez et al. 2021). Aquaculture artificial substrates may serve as stepping stones, offering novel niches for opportunistic colonizers, including NIS, favoring their dispersal (De Mesel et al. 2015) and supplying substrate to establish other NIS (Rius et al. 2011; Png-Gonzalez et al. 2021).

In this context, the current study examined samples of 10 bryozoan species stemming from NIS monitoring surveys conducted on artificial substrates along the south coast of the Madeira Archipelago in four recreational marinas and two offshore aquaculture farms. This research resulted in the description of two new bryozoan species and four new records for the archipelago.

## Materials and methods

**Fig. 1:**
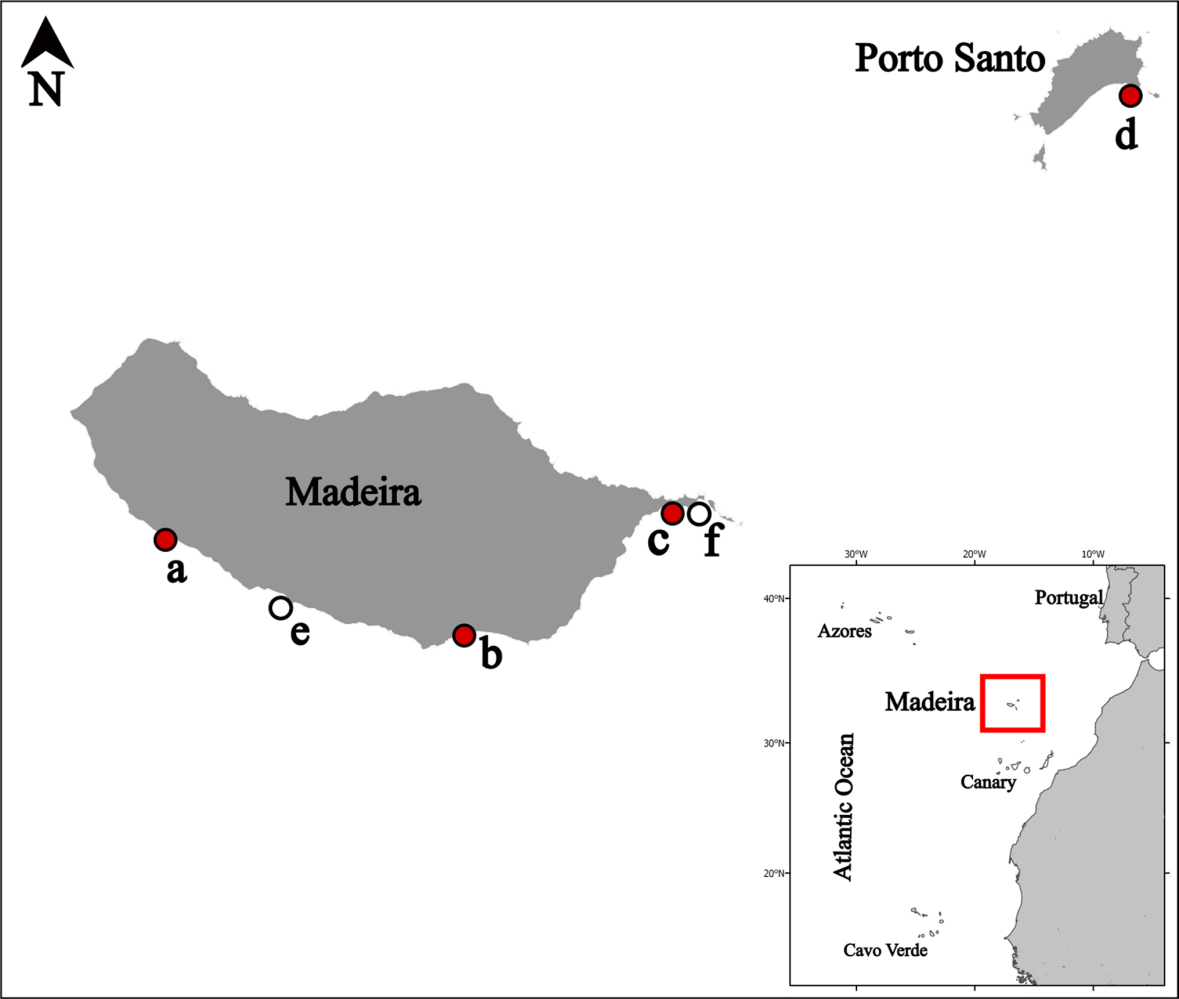
Madeira Archipelago with the location of the four marinas along the south coast of both Madeira and Porto Santo Islands shown by a red circle; a—Calheta (CA); b—Funchal (FX); c—Quinta do Lorde (QL), and; d—Porto Santo (PS); two aquaculture facilities shown by a white circle; e—Campanário (CAM) and; f—Caniçal (CAN)

The bryozoan samples stem from several NIS monitoring surveys conducted along the south coast of the Madeira Archipelago (Fig. 1), particularly on artificial settlement panels deployed within four recreational marinas: Calheta (CA, 32°43′ N, 17°10′ W), Funchal (FX, 32°38′ N, 16°54′ W), Quinta do Lorde (QL, 32°44′ N, 16°42′ W), and Porto Santo Island (PS, 33°03′ N, 16°18′ W), between 2013 and 2019 (Fig. 1a–d). In addition, this includes samples collected during 2018 from two offshore aquaculture farms growing gilthead sea bream (*Sparus aurata* Linnaeus, 1758), Campanário (CAM, 32°39′ N, 17°3′ W) and Caniçal (CAN, 32°44′ N, 16°41′ W) (Fig. 1e, f). Following the design employed by Canning-Clode et al. (2011) and Ramalhosa et al. (2014), at each sampling site polyvinylchloride (PVC) experimental settling panels (14 × 14 × 0.3 cm) were deployed attached to a brick, They were positioned horizontally and facing downwards to favor the settlement of macro-invertebrates rather than macro-algae and hung at approximately 1 m depth from pontoons inside the four marinas and in the external float of seabream offshore cages at each aquaculture farm. Settlement plates were retrieved after three months to collect representative samples. Fouling communities from settlement plates were initially observed with a stereomicroscope (Leica S8 APO), and digital photographs of specimens were taken using an Olympus STYLUS TG-4 camera. Bryozoan specimens were first preserved in 95% ethanol and later examined with a stereomicroscope Leica MZ12. Colony fragments were selected and dried, and SEM photographs of uncoated material were taken using an FEI Inspect S50 SEM at the University of Vienna, Austria. Some samples were cleaned, and when necessary, fragments were bleached with sodium hypochlorite to remove organic material before SEM examination. The SEM was used with a back-scattered electron detector in low vacuum mode. Zooidal measurements were taken from the SEM images using the software ImageJ® (http://rsbweb.nih.gov/ij). In addition, historical specimens from the Norman Collection stored in the Museu Municipal de História Natural in Funchal (MMF), Madeira Island, Portugal, were also examined. Furthermore, type specimen of *Celleporaria brunnea* (Hincks, 1884) from the Natural History Museum of London (NHMUK) and specimens of this species from the Iberian Peninsula and Italy were studied for comparison. Finally, new specimens collected during the present study were deposited in MMF and the Natural History Museum of the University of Santiago de Compostela (MHNUSC) collections.

## Results

**Phylum Bryozoa **Ehrenberg, 1831

**Class Stenolaemata **Borg, 1941

**Order Cyclostomatida **Busk, 1852

**Family Crisiidae **Johnston, 1838

**Genus Crisia **Lamouroux, 1812

***Crisia noronhai* sp. nov.** Souto, Ramalhosa & Canning-Clode

https://zoobank.org/23D69F5B-1684-4167-84C2-2D37C8985884

(Fig. 2, Table 1)

**Fig. 2:**
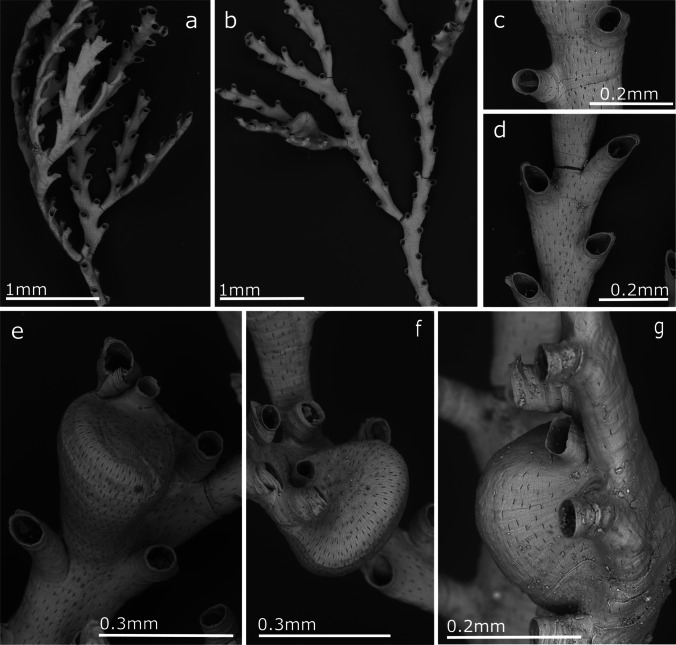
*Crisia noronhai* sp. nov; **a, b** General view of colonies; **c** Orifices of two autozooids; **d** Autozooids and articulation portion of erect colony; **e–g** Gonozooids with ooeciostoma

**Table 1:** Measurements (in mm) of *Crisia noronhai* sp. nov.

	Mean	SD	Minimum	Maximum	*N*
Internode width	0.233	0.0283	0.190	0.274	10
Internode length	1.605	0.3680	1.09	2.131	8
Zooid length	0.355	0.0411	0.281	0.486	46
Zooidal peristome length	0.124	0.0242	0.063	0.171	40
Peristomial aperture diameter	0.068	0.0076	0.052	0.080	41
Gonozooid length	0.439	0.0274	0.401	0.476	8
Gonozooid width	0.284	0.0256	0.255	0.324	8

*SD* standard deviation, *N* number of measurements

*Crisia eburnea*, Norman 1909: 277.

**Material examined**

**Holotype**– MMF48464: 13/01/2014, Funchal, two dry fragments from one specimen in a SEM stub; **Paratypes**– MMF48461, 44,786, 48,462: 11/10/2013, Quinta do Lorde; MMF48463: 15/10/2013, Calheta; MMF48465: 29/07/2014, Quinta do Lorde; MMF47068: 26/01/2015, Quinta do Lorde; MMF48466, 48,467: 30/10/2018, Caniçal; MMF48468: 48,469: 05/11/2018, Campanário. **Other material**–MMF44688 (Noronha 1907?, Funchal, Lido), MMF44775 (Noronha 1907?, Bay of Funchal).

**Etymology**

This species is dedicated to Adolfo César de Noronha (1873–1963), an enthusiastic naturalist and avid promoter of the Museu de História Natural do Funchal. For years, Noronha collected specimens of bryozoans from Madeira Archipelago that were included in Norman’s collection.

**Description**

Erect colonies forming dense tufts, between 1 and 2 cm in height, attached to the substrate by rhizoids formed from the basal branches of the colony. Branches, lightly incurving. Internodes formed by 7 and 16 zooids, with 9–11 zooids being the most common. Each internode has from 1 to 3 lateral branches; the first branch commonly forms at level of first zooid of an internode when two or three branches develop from the same internode, but from the third zooid when only one branch derive from one internode. Subsequent branches alternate on the opposite side from the parental internode, without a clear pattern in the sequence of appearance in the zooid position. Internodes articulate with joints ranging from colourless (in young portions of the colony) to light brown (old colony parts). Autozooids tubular, with a peristome around one-third of zooid length. Orifice facing forward, circular; in some cases, presenting a pointed process lateral to the orifice. The frontal surface is densely covered by pseudopores, without differences in their density between gonozooids and autozooids (average 1443.5 pseudopores/mm^2^). Several tubular rhizoids support the colony over the substrate, forming from the basal part of the first branches, never present in the younger portions of the colony. Gonozooids pyriform, rather prominent in profile, proportionally broad and reaching maximum width at the end of its length, with the distal part flat or slightly concave. Oeciostoma round, smaller than the autozooid’s aperture.

**Remarks**

The species described here presents morphological characteristics that differ from the known species belonging to the genus *Crisia*. The morphologically closest related species is *Crisia eburnea* (Linnaeus, 1758). Nevertheless, *C. eburnea* presents shorter internodes with fewer zooids (5–7 according to Kluge 1975 and Hayward and Ryland 1985), only one branch per internode, pyriform gonozooid, and a lower pore density on the zooidal wall, *Crisia noronhai* sp. nov., in turn, features longer internodes and gonozooids that are wider and whose distal part is concave. *Crisia eburnea* is widely distributed along the eastern Atlantic Ocean from Norway to the Mediterranean Sea. It was recorded by Norman (1909) from Madeira. Nevertheless, specimens collected by Noronha identified by Norman as *C. eburnea* and stored in the MMF, examined during the present study proved to be conspecific with *C. norhonai*..

d'Hondt (1988) described from Guipuzcoa (N Spain) *C. eburnea* subsp. *Harmelini*. This subspecies has a larger number of zooids per internode (8–18, more usually 13–14), closer to the observed zooid number in *C. noronhai* sp. nov. Nevertheless, a maximum of two branches are formed from one internode, and the morphology of the gonozooid is significantly different, being more elongated and narrower than in *C. noronhai* sp. nov*.*

*Crisia noronhai* sp. nov*.* is also similar to the Artic species, *Crisia eburneodenticulata* Smitt ms in Busk 1875, presenting a similar branching pattern. According to Kluge (1975), this species commonly has 11–13 zooids per internode and up to 20, but whose autozooids are almost entirely adnate, with a very short peristome and gonozooids that are pyriform but distally convex, attaining their maximum width at one-third of their lengths.

Finally, this study possibly confirms the identity of the previously unknown *Crisia* sp. samples collected since 2013 in all marinas in the Madeira Archipelago, as shown in Ramalhosa et al. (2019). Importantly, early records of this species date back to the 1900s until the present time.

***Crisia* sp. aff. *elongata ***Milne-Edwards, 1838

(Fig. 3, Table 2).

**Fig. 3:**
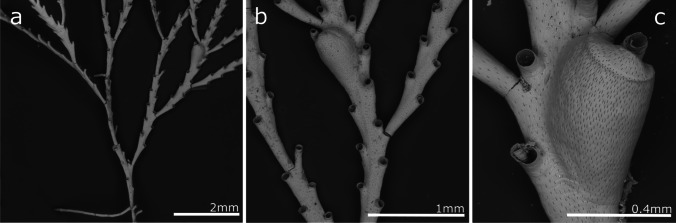
*Crisia* aff. *elongata*; **a** Colony; **b** Close up of colony branching, autozooid orifices, and gonozooid; **c** Gonozooid

**Table 2:** Measurements (in mm) of *Crisia* sp. aff. *elongata*

	Mean	SD	Minimum	Maximum	*N*
Internode width	0.255	0.0308	0.197	0.283	6
Internode length	2.442	0.3491	1.970	2.841	6
Zooid length	0.560	0.1045	0.445	0.962	29
Peristomial aperture diameter	0.076	0.0065	0.063	0.091	29
Gonozooid length	0.689	0.0372	0.647	0.717	3
Gonozooid width	0.356	0.0418	0.294	0.382	4

*SD* standard deviation, *N* number of measurements

?*Crisia elongata* Milne-Edwards, 1838: 203.

?*Crisia elongata*: Norman, 1909: 277; Harmer, 1915: 96, Pl. VII, figs. 1-8.

**Material examined**

MMF48470: 09/08/2013, Calheta; MMF48471: 14/10/2013, Funchal; MMF48472: 13/01/2014, Funchal; MMF45060: 04/08/2014, Calheta; MMF47045: 06/02/2015, Funchal.

**Description**

Colony erect, forming bushy tufts 1.5–2.5 cm high, comprising jointed biserial branches. Internodes very variable in size, including from 4 to 18 zooids that are generally longer in the distal parts and shorter in the basal part of the colony (and occasionally in medial parts of some colonies as well).. Generally, one branch is presented by internodes and two branches in the internodes with gonozooids. The joints are black. Autozooids are tubular, with a very short peristome and a circular orifice facing forward. Gonozooids pyriform with the broader portion distally, ooeciopore slit-like, without ooeciostomal rim. Autozooidal orifices adjacent to gonozooids are slightly narrower than when no gonozooids are present. The gonozooid frontal wall is densely porous, with an average of 1460 pseudopores/mm^2^ in autozooids and 3520 pseudopores/mm^2^ in gonozooids. Colonies attach to the substrate by tubular rhizoids forming from the basal part of the first branches.

**Remarks**

Unlike our material, *Crisia elongata* previously recorded in Madeira by Norman (1909) was collected in deeper waters, between 73 and 128 m deep (40–70 fathoms), and indicated as an uncommon species. Norman’s original specimen was not found, so we cannot be certain if it was conspecific with our material. In contrast, colonies from Ramalhosa et al. (2019) recorded as Crisia sp. from marinas of Calheta and Funchal, were partly examined and identified as *Crisia* sp. aff. *elongata*.

*Crisia elongata* was described from the Red Sea (Milne-Edwards 1838) and fossil and recent specimens were later recorded on several occasions. Currently, *C. elongata* is considered a widespread species in the Indian and Pacific Oceans (Harmer 1915; Lagaaij 1959; Winston 1986; Gordon 1989; Ziko et al. 2012; Chae et al. 2020), but also in the Mediterranean Sea (Rosso and Di Martino 2016). Madeiran specimens show some differences compared to specimens described from the Pacific, which have a fewer zooids per internode and proportionally longer gonozooids. Note, however, that differences also exist between the specimens figured by Gordon (1989) from Samoa, Winston (1986) from Panama, and those by Chae et al. (2020) from Korea; even twin gonozooids are described. These variations call for a re-description of *C. elongata* type material, and no formal identification is assumed for Madeiran specimens possibly representing a new species.

**Class Gymnolaemata **Allman, 1856

**Order Ctenostomatida **Busk, 1852

**Family Vesiculariidae **Hincks, 1880

**Genus *Amathia ***Lamouroux, 1812

***Amathia madeirensis* sp. nov.** Souto, Ramalhosa & Ferrario.

https://zoobank.org/7BE3C5D7-F22F-450C-A664-B6B3A91E877A

(Fig. 4, Table 3)

**Fig. 4:**
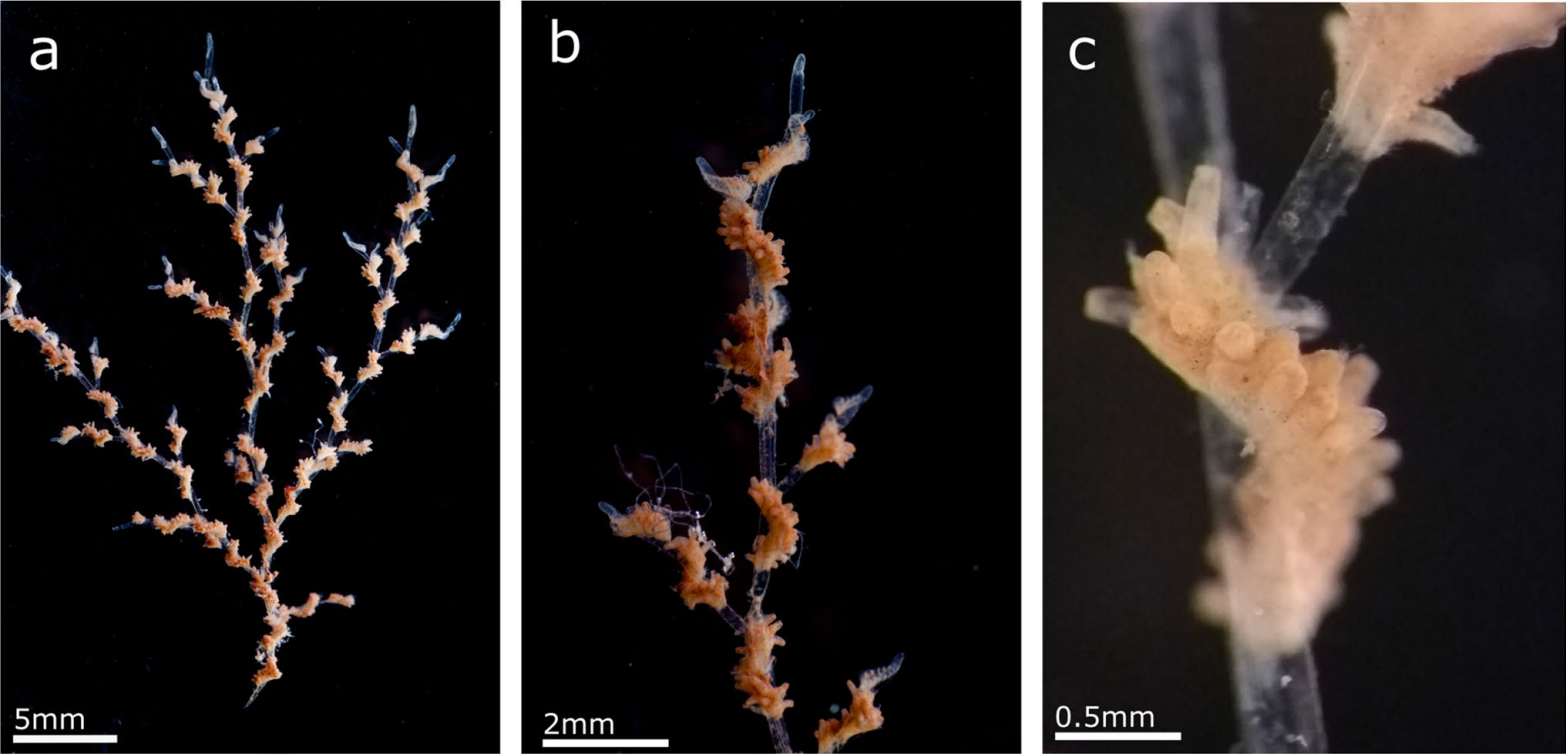
*Amathia madeirensis* sp. nov; **a** General view of colony; **b** Growing part of branches in a colony; **c** Group of autozooids and branch

**Table 3:** Measurements (in mm) of *Amathia madeirensis* sp. nov.

	Mean	SD	Minimum	Maximum	*N*
Stolon length	2.368	0.2632	1.989	2.711	12
Stolon width	0.267	0.0534	0.178	0.385	22
Zooidal group length	1.400	0.1898	1.111	1.772	12
Zooid length	0.463	0.0463	0.399	0.548	14
Zooid width	0.132	0.0187	0.091	0.158	14

*SD* standard deviation, *N* number of measurements

**Material examined**

**Holotype**– MMF47097, 13/11/2017, Calheta.

**Etymology**

The name, *madeirensis*, alludes to the Madeira Archipelago, where the specimen was collected.

**Description**

Colony arborescent, branching, up to 5 cm high, forming dense light brown coloured tufts. Each branch is formed by cylindrical autozooid-bearing kenozooids separated by transverse septa. Branching is lateral, directly before the septa in all kenozooids. New stolons budded laterally at 45–60° to the stolonal axis. In-line stolons may be lightly deflected after ramification, giving the impression of a dichotomy. Kenozooids bear an extended group of 26–30 autozooids, occupying between half and two-thirds of the length of the kenozooids in their distal part. Autozooid groups are twisted, undergoing a 180–270° torsion around the kenozooid. The spiral direction is constant within a colony, but may differ between colonies, being either “clockwise” or “anticlockwise”.. The last zooid in a group and the first of the succeeding group are almost in the same plane.

Autozooids budded at the growing tips of each branch as small vesicles arranged in two series along the spiral. Autozooids cylindrical, but with subquadrangular apertures at the truncated distal ends. Autozooids closely packed, but not attached to eachother along their distal halves. Polypide with eight tentacles.

**Remarks**

Specimens from Madeira are morphologically closely related to *Amathia pustulosa* (Ellis & Solander, 1786). That species also has long zooids groups of up to 30 zooids per group. Nevertheless, the lateral walls of *A. pustulosa* zooids completely free, not joined to the adjacent zooids in the group (Prenant and Bobin 1956; Souto et al. 2010), whereas in *A. madeirensis* sp. nov. the autozooids are closely packed with their proximal halves joined to the adjacent zooids. Moreover, *A. pustulosa* presents many rhizooids that attach the colony to the substrate and generate new erect portions (Souto et al. 2010), which were not observed in the Madeiran specimens. *Amathia pustulosa* also has series of stolonar kenozooids missing lateral branches, whereas all kenozooids have lateral branches in *A. madeirensis* sp. nov.

Two other species, *Amathia brasilensis* Busk, 1886 and *Amathia distans* Busk, 1886, re-described by Fehlauer-Ale et al. (2011), have a similar colony morphology. Both species feature a very variable number of zooids per group, between 8 and 18 pairs in *A. brasilensis*, and 9–19 pairs in *A. distans*. Moreover, in both species the group of zooids form a complete spiral around the stolon and with the zooids mostly unjoined*.* Finally, both species have shorter and thinner stolons than *A. madeirensis* sp. nov., and *A. distans* has yellow spots in both stolons and autozooids.

**Order Cheilostomatida **Busk, 1852

**Genus *Scruparia ***Oken, 1815

*Scruparia ambigua* (d’Orbigny, 1841)

(Fig. 5, Table 4).

**Fig. 5:**
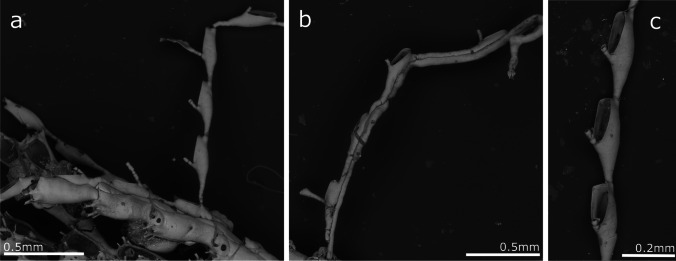
*Scruparia ambigua*; **a** Erect and encrusting part of a colony growing on *Cradoscrupocellaria bertholletii*; **b** Encrusting part of one colony growing on a rhizoid of *C. bertholletii*; **c** Autozooids

**Table 4:** Measurements (in mm) of *Scruparia ambigua*

	Mean	SD	Minimum	Maximum	*N*
Autozooid length	0.379	0.0286	0.342	0.425	11
Autozooid width	0.111	0.0052	0.104	0.116	6
Opesia length	0.170	0.0081	0.158	0.179	11
Opesia width	0.095	0.0162	0.069	0.120	11

*SD* standard deviation, *N* number of measurements

*Eucratea ambigua* d’Orbigny, 1841:pl. 3, figs. 13–17.

*Scruparia ambigua*: Hastings, 1941: 465; Hayward and Ryland, 1998,108, Fig. 19.

**Material examined**

MMF48474: 30/10/2018, Caniçal, on *Cradoscrupocellaria bertholletii*.

**Description**

Colony with encrusting and erect parts formed by uniserial chains of zooids. Encrusting uniserial portions with autoozoids budded from each zooid's distal part, and branches formed by lateral budding. Erect part arises from the frontal wall of the repent autozooids, forming uniserial chains of autozooids with branches always formed by frontal budding. Autozooids are slender and long, with their half distal parts being wider. Frontal oval opesia takes up about half of autozooid length and is located in the distal portion of the zooid. No brood chambers were observed in the Madeira specimens, but have benn described as bivalve brood chambers on dimorphic zooids (Zabala 1986; Hayward and Ryland 1998; Hayward and McKinney 2002; De Blauwe 2009).

**Remarks**

*Scruparia ambigua* is a widespread species recorded worldwide except for polar regions (Hayward and Ryland 1998). In general, this species is grows on bryozoans, algae, hydroids, stones and shells in shallow waters, not deeper than 50 m (Hayward and Ryland 1998), but also in artificial substrates (Ryland 1965; Beukhof et al. 2016; McCuller and Carlton 2018; Coolen et al. 2020). However, it was not previously recorded in the Madeira Archipelago. Here we present the first Madeiran record of this species, which was observed on artificial panels and on erect bryozoans and hydroids.

**Family Bugulidae **Gray, 1848

**Genus *Bugula ***Oken, 1815

***Bugula ingens***Vieira et al., 2012

(Fig. 6, Table 5).

**Fig. 6:**
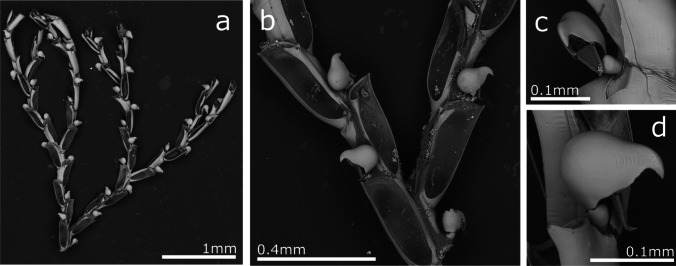
*Bugula ingens*; **a** General view of colony; **b** Zooids and avicularia at a branching point; **c, d** Avicularia

**Table 5:** Measurements (in mm) of *Bugula ingens*

	Mean	SD	Minimum	Maximum	*N*
Autozooid length	0.408	0.0430	0.346	0.485	12
Autozooid width	0.117	0.0146	0.078	0.139	12
Frontal membrane length	0.318	0.0148	0.297	0.339	12
Frontal membrane width	0.085	0.0136	0.047	0.098	12
Avicularia length	0.126	0.0040	0.117	0.131	9

*SD* standard deviation, *N* number of measurements

*Bugula ingens* Vieira et al., 2012: Figs. 7B, 10B, D and F.

**Material examined**

MMF48475: 05/11/2018, Campanário.

**Description**

Colony erect, with biserial branches of alternating and slightly rotated zooids simulating a uniserial pattern. Colony branches with 3 to 5 zooids between branches. Zooids are slightly calcified and elongated, with the frontal membrane occupying around one-third of the total zooid length. Distal corners of the zooids are slightly pointed but without spines. Avicularia pedunculate, present in all zooids, attached by a tubular cuspidate peduncle situated close to the proximal margin of the zooid and situated on the external wall of the zooid, in distal position to the previous zooid. Avicularia rounded with a hooked rostrum. Rostrum lateral edge irregular but not denticulate. Ovicell and ancestrula were not observed in the Madeiran specimens.

**Remarks**

Specimens found in Madeira are morphologically a member of the *B. uniserialis-*group (Vieira et al. 2012; Fehlauer-Ale et al. 2015). The specimens studied here agree with the description and biometrics of *B. ingens* without many differences*.* This species was described for only one locality, Meros, Ilha Grande, Angra dos Reis, Brazil (Vieira et al. 2012) and was not re-recorded after its original description. *Bugula ingens* is very similar to *B. gnoma* Vieira et al. 2012 and *B. rochae* Vieira et al. 2012, but as those authors pointed out, it is quite distinct from these two species by the conspicuously large avicularia.

Here we present the first record of this species on Madeira Island and outside its type locality in Brazil. The scarce of data about this species make difficult to know if this species can be considered as native or alien on the archipelago.

**Family Candidae **d’Orbigny, 1851

**Genus *Cradoscrupocellaria ***Vieira et al., 2013

***Cradoscrupocellaria bertholletii* (**Audouin, 1826**)**

(Fig. 7, Table 6).

**Fig. 7:**
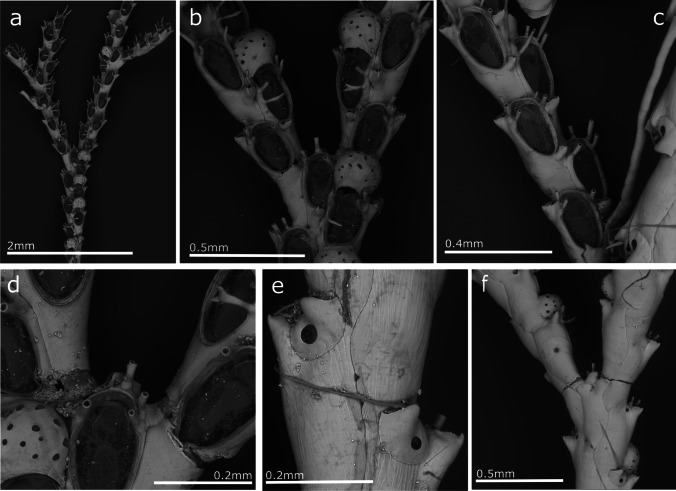
*Cradoscrupocellaria bertholletii*; **a** General view of colony; **b** Zooids on branches and ovicells; **c** Zooids with a view of lateral avicularia; **d** Spines distribution on zooid of the branch; **e** Vibracularia on dorsal wall; **f** Dorsal view of branching

**Table 6:** Measurements (in mm) of *Cradoscrupocellaria bertholletii*

	Mean	SD	Minimum	Maximum	*N*
Autozooid length	0.417	0.0316	0.346	0.478	27
Autozooid width	0.176	0.0149	0.144	0.202	27
Opesia length	0.254	0.0235	0.222	0.302	27
Opesia width	0.139	0.0103	0.116	0.157	27
Ovicell length	0.170	0.0105	0.157	0.184	11
Ovicell width	0.201	0.0101	0.187	0.214	11
Lateral avicularium length	0.069	0.0092	0.055	0.085	23
Vibracular chamber length	0.121	0.0109	0.100	0.152	19
Vibracular chamber width	0.101	0.0142	0.073	0.123	19

*SD* standard deviation, *N* number of measurements

*Acamarchis bertholletii* Audouin, 1826: 241; Savigny, 1817: pl. 11, figs. 3.1–3.5.

*Scrupocellaria bertholletii*: Hincks, 1886: 258, pl. 9, figs. 1–2; Ramalho, 2006: 127, fig. 28; *Cradoscrupocellaria bertholletii*: Vieira et al. 2013: 7, figs. 2–3.

**Material examined**

MMF49208, 49,209: Calheta, 15/10/2013; MMF49210, 49,211, 49,212: Porto Santo, 23/10/2013; MMF48473: 30/10/2018, Caniçal; MMF48476: 05/11/2018, Campanário.

**Remarks**

*Cradoscrupocellaria bertholletii* was recently redescribed by Vieira et al. (2013), and new specimens collected during this study in Madeira correspond to the previous descriptions given for this species. Many records of this species exist worldwide, from shallow waters in New Zealand, Suez Canal, the Mediterranean and Brazil (Vieira et al. 2013). It was recorded previously in Madeira by Norman (1909). Vieira et al. (2013), in a mistake (Vieira, personal communication), write that Norman’s record could not be assigned to this species. However, its presence in Madeira was included based on a sample identified by Norman and labelled with the unpublished name *Scrupocellaria bertholletii* var. *aperta*, identified by Vieira et al. (2013) as *C. bertholletii*. Nevertheless, the description and figures presented by Norman (1909) agree with the characteristics of this species. In the absence of the original specimens described by Norman (1909), we consider his record as the first record of *C. bertholletii* for Madeira in the present work. More recently, the presence of this species in marinas around Madeira was recorded by Canning-Clode et al. (2013a) and Ramalhosa et al. (2019) as non-indigenous, but its status has recently been changed to cryptogenic (Castro et al. 2020). In the current study, its species is confirmed on the artificial substrate, and new data and SEM figures are provided.

***Cradoscrupocellaria insularis ***Vieira et al., 2013

(Fig. 8, Table 7).

**Fig. 8:**
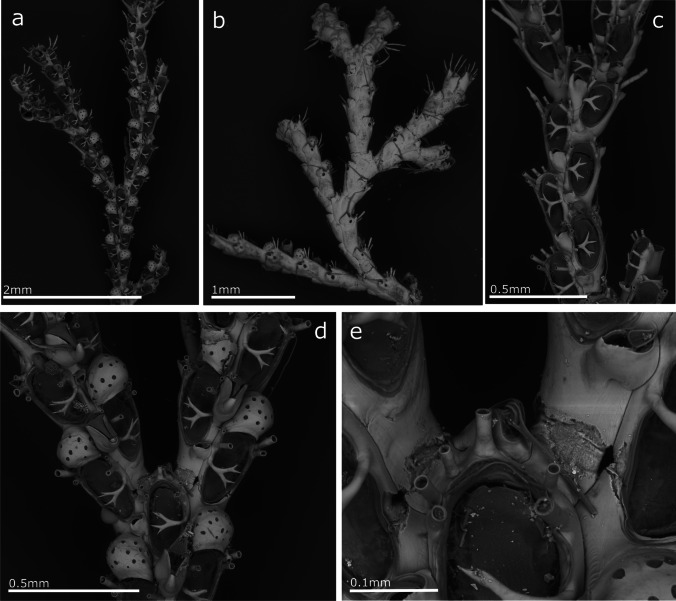
*Cradoscrupocellaria insularis*; **a** Frontal view of a colony part; **b** Dorsal view of a colony part; **c** Autozooids; **d** Ovicellate autozooids and large frontal avicularia on a branching part of colony; **e** Spine distribution on axial zooid

**Table 7:** Measurements (in mm) of *Cradoscrupocellaria insularis*

	Mean	SD	Minimum	Maximum	*N*
Autozooid length	0.408	0.0309	0.367	0.453	15
Autozooid width	0.180	0.0180	0.149	0.210	15
Opesia length	0.276	0.0297	0.218	0.324	15
Opesia width	0.134	0.0121	0.117	0.165	15
Ovicell length	0.161	0.0103	0.145	0.182	12
Ovicell width	0.179	0.0128	0.155	0.193	12

*SD* standard deviation, *N* number of measurements

*Cradoscrupocellaria insularis* Vieira et al., 2013: Fig. 13.

**Material examined**

MMF48477: 30/10/2018, Caniçal.

**Remarks**

*Cradoscrupocellaria insularis* was recently described based on historical specimens collected in Cape Verde, besides specimens recorded in Madeira in 1857, identified initially as *Scrupocellaria bertholletii* and currently stored in the NHMUK (Vieira et al. 2013). New specimens collected on artificial panels during this work agree with the morphological description of *C. insularis*. This new record confirms the species' current presence in Madeira.

**Genus *Scrupocaberea ***Vieira et al., 2014

***Scrupocaberea maderensis* (**Busk, 1860**)**

(Fig. 9, Table 8).

**Fig. 9:**
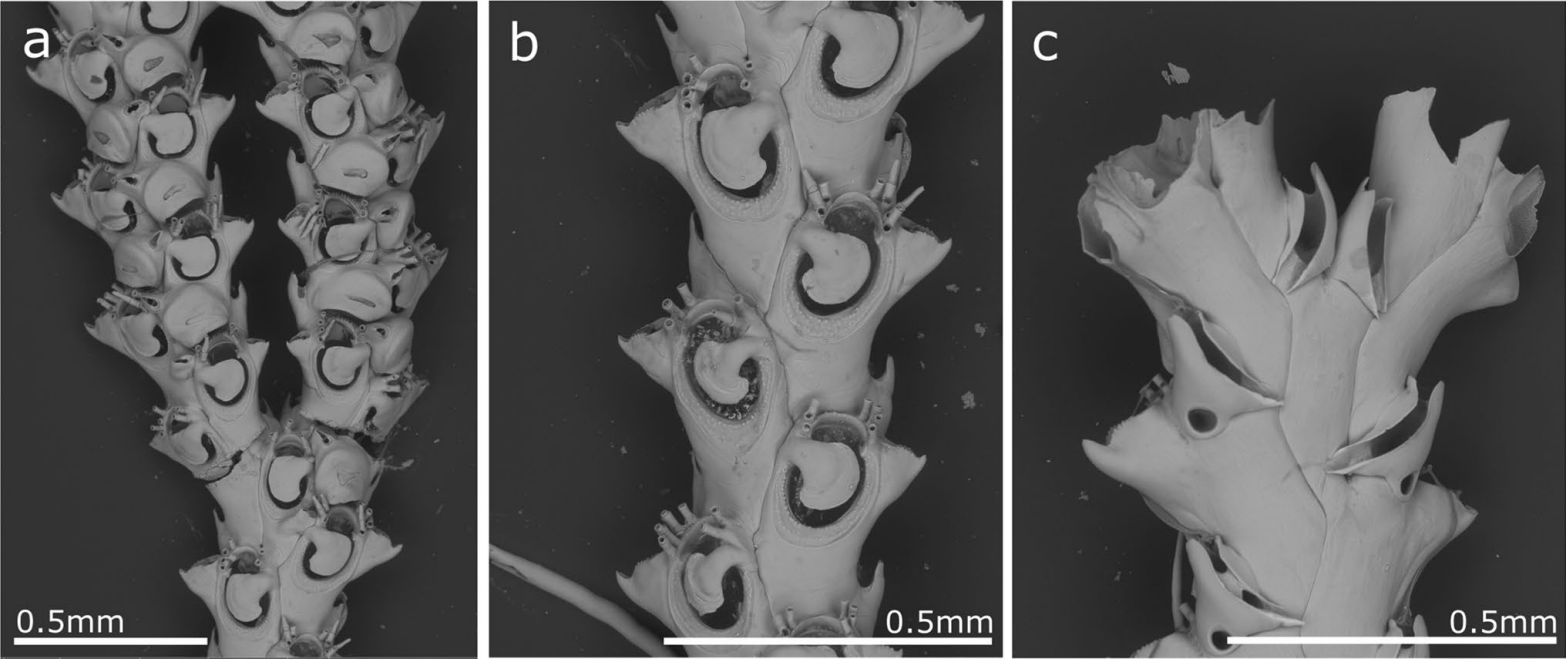
*Scrupocaberea maderensis*; **a** Frontal view of a bifurcation with ovicellate zooids; **b** Frontal view of zooids with details of scuta and lateral avicularia; **c** Dorsal view of colony with details of vibracularia

**Table 8:** Measurements (in mm) of *Scrupocaberea maderensis*

	Mean	SD	Minimum	Maximum	*N*
Autozooid length	0.365	0.0207	0.313	0.390	20
Autozooid width	0.173	0.0124	0.139	0.190	20
Opesia length	0.193	0.0127	0.166	0.216	20
Opesia width	0.119	0.0067	0.106	0.129	20
Scutum length	0.119	0.0120	0.098	0.139	20
Scutum width	0.112	0.0088	0.091	0.124	20
Lateral avicularium length	0.113	0.0213	0.073	0.146	14
Ovicell length	0.135	0.0180	0.108	0.160	11
Ovicell width	0.170	0.0144	0.145	0.193	11
Vibracular chamber length	0.196	0.0118	0.182	0.215	8
Vibracular chamber width	0.167	0.0146	0.135	0.180	8

*SD* standard deviation, *N* number of measurements

*Scrupocellaria maderensis* Busk, 1860: 280;

*Scrupocellaria maderensis,* Busk, 1861: 77, pl. 32, fig. 1; Norman 1909: 284. *Scrupocaberea maderensis*: Vieira et al. 2014: 17, figs. 15A–C; Souto et al. 2015: figs. 18–20.

**Material examined**

MMF47013: 24/08/2015, Quinta do Lorde; MMF47034: 17/12/2015, Quinta do Lorde; MF47046: 02/04/2016, Quinta do Lorde; MMF48478: 04/10/2018, Quinta do Lorde.

**Remarks**

*Cradoscrupocellaria maderensis* was described and figured from Madeira (Busk, 1860, 1861) as *Scrupocellaria maderensis* and recently transferred to the genus *Scrupocaberea* (Vieira et al. 2014). This species has been reported to be widespread in tropical and subtropical waters worldwide (Harmer 1926; Tilbrook 2006; Vieira et al. 2014), although the identity of these records should be revised (Vieira et al. 2014). In Madeira, this is the first record of *C. maderensis* on artificial substrates after the recent finding on rocky substrates at 11 m depth (Souto et al. 2015).

**Genus *Tricellaria*** Fleming, 1828

***Tricellaria inopinata*** d'Hondt and Ambrogi, 1985

(Fig. 10, Table 9).

**Fig. 10:**
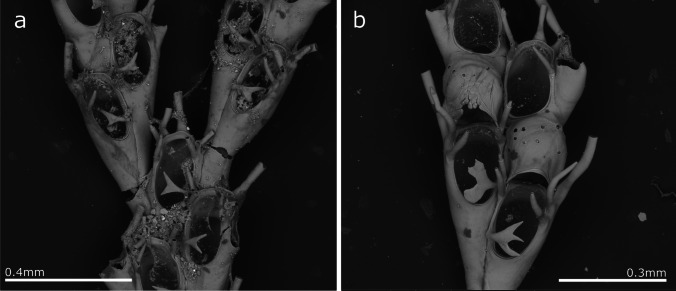
*Tricellaria inopinata*; **a** Zooids at a bifurcation; **b** Ovicellate zooids

**Table 9:** Measurements (in mm) of *Tricellaria inopinata*

	Mean	SD	Minimum	Maximum	*N*
Autozooid length	0.477	0.0542	0.404	0.549	12
Autozooid width	0.153	0.0125	0.134	0.173	12
Opesia length	0.238	0.0158	0.214	0.261	12
Opesia width	0.120	0.0119	0.102	0.141	12
Ovicell length	0.173	0.0109	0.163	0.187	4
Ovicell width	0.174	0.0105	0.166	0.189	4

*SD* standard deviation, *N* number of measurements

***Tricellaria inopinata*** d'Hondt and Ambrogi, 1985: 35–46, figs. 2, 3.

**Material examined**

MMF48479, 48,480: 30/10/2018, Caniçal.

**Remarks**

Firstly described based on specimens collected in the lagoon of Venice (d'Hondt and Ambrogi 1985), *T. inopinata* is currently thought to have originated on the Pacific coast of North America (Dyrynda et al. 2000; De Blauwe and Faasse 2001). After its description, this species was recorded as NIS around the globe, from the north of Japan to Taiwan, Australia, New Zealand, and the Mediterranean Sea (Occhipinti Ambrogi and d'Hondt 1994). This species is also present in the North-East Atlantic Ocean, where it has shown a rapid expansion over the past few years (e.g. De Blauwe and Faasse 2001, Bishop et al. 2015, Porter et al. 2015, 2017; Reverter-Gil et al. 2019), even reaching the Azores (Micael et al. 2016). Nominal records of *T. inopinata* from Madeira were presented by Ramalhosa et al. (2019) as non-indigenous on artificial substrates from all marinas. In this study, the species' identity is confirmed for Madeira, and new data and SEM figures are provided.

**Family Lepraliellidae **Vigneaux, 1949

**Genus *Celleporaria ***Lamouroux, 1821

**Fig. 11:**
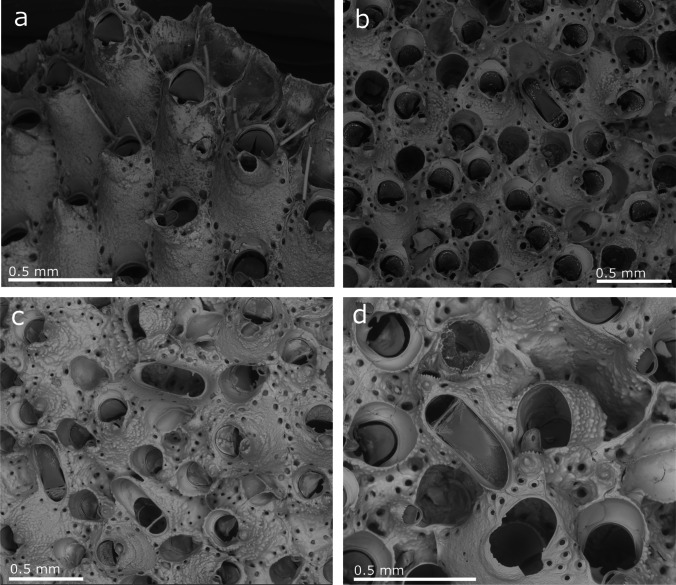
*Celleporaria brunnea*; **a** Colony margin where zooids still bear oral spines; **b, c** General views of colonies with spatulate avicularia; **d** Spatulate avicularia and ooecia

**Fig. 12:**
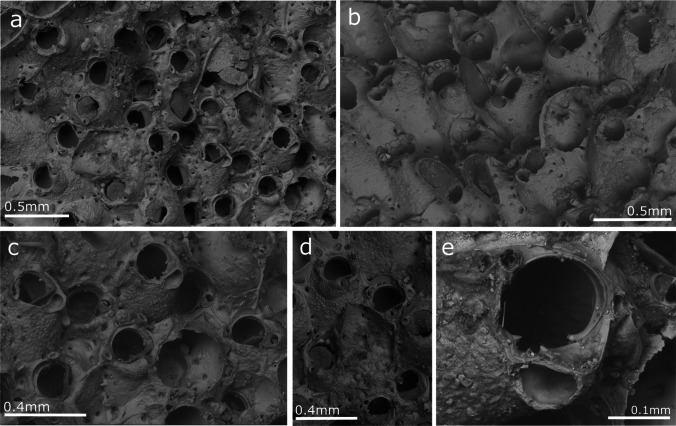
*Celleporaria brunnea*. Type specimen (NHMUK-1886.3.6.33); **a** General view of colony;**b** View of zooids with oral spines and spatulate avicularia; **c, d** Zooidal orifices. E. Detail of oral orifice

**Fig. 13:**
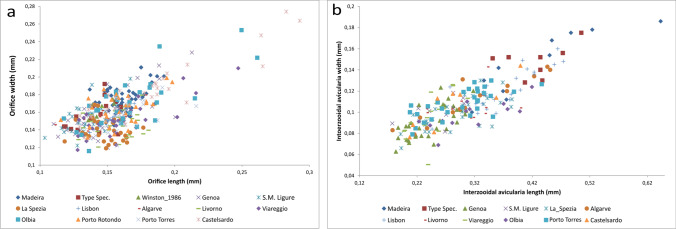
Morphometric comparison of *C. brunnea*; **a** Comparison of orifice size of specimens from different localities; **b** Comparison of interzooidal spatulate avicularia of specimens from different localities

**Fig. 14:**
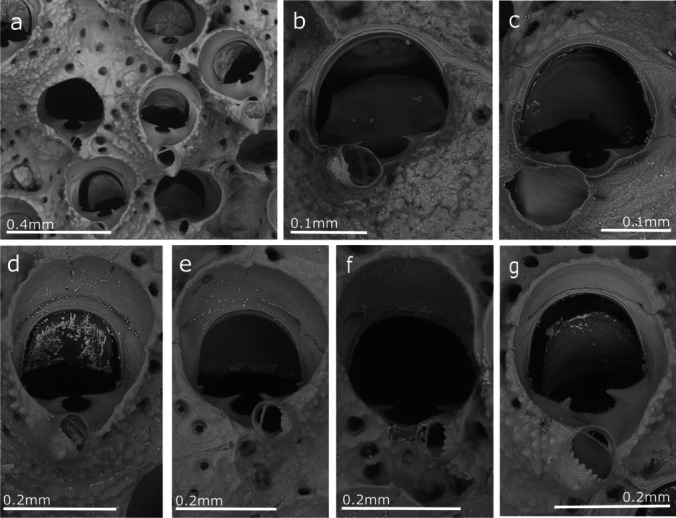
*Celleporaria brunnea*. Visual comparison of the intravariability in the orifice morphology from two colonies collected in Madeira; **a, d–g** colony MMF48483 and; **b, c** colony MMF48481

***Celleporaria brunnea* (**Hincks, 1884**)**

(Figs. 11, 12, 13, and 14, Table 10)

**Table 10:** Measurements (in mm) of specimens from Madeira of *Celleporaria brunnea*

	Mean	SD	Minimum	Maximum	*N*
Autozooid length	0.678	0.0877	0.534	0.89	35
Autozooid width	0.363	0.0537	0.258	0.5	35
Orifice length	0.163	0.0117	0.137	0.192	48
Orifice width	0.174	0.0144	0.145	0.211	48
Suboral avicularia length	0.080	0.0140	0.063	0.095	16
Suboral avicularia width	0.062	0.0089	0.049	0.075	16
Spatulate avicularia length	0.463	0.1062	0.315	0.643	10
Spatulate avicularia width	0.154	0.0324	0.100	0.186	10

*SD* standard deviation, *N* number of measurements

*Cellepora brunnea* Hincks, 1884, p. 56; O'Donoghue and O'Donoghue, 1926, p. 21.

*Holoporella brunnea*: Hastings, 1929, p. 731, p. 16, fig. 7, 108–110; Osburn, 1952, p. 496, pl. 62, figs. 10–12; Soule, 1961, p. 33; Soule and Soule, 1965, p. 38, fig. 13, 14.

*Celleporaria brunnea*: Winston, 1986, p. 12, figs. 19–22; Soule et al. 1995, p. 267, fig. 101; Koçak, 2007, p. 192, fig. 2 A-D; Seo and Min 2009, p. 29, fig. 7; Canning-Clode et al. 2013b, fig. 2; Lodola et al. 2015, p. 265, figs.3–5; Lezzi et al. 2015, p. 1, fig. 2–3; McCuller and Carlton 2018, p. 153, fig. S18B.

*Celleporaria* sp. aff. *brunnea*: Harmelin, 2014, p. 316, fig. 6.

**Material examined**

MMF48482: 22/06/2018, Quinta do Lorde; MMF48481: 26/02/2019, Quinta do Lorde; MMF48483: 06/06/2019, Porto Santo; MHNUSC-698: Marina de Cascais, Portugal, 13/05/2016; MHNUSC-699: Ocean Revival, Algarve, Portugal, 12/07/2014.

**Description**

Colony encrusting, irregularly shaped, unilaminar to multilaminar depending on the development stage. Zooids white to grey, but with the operculum and mandible of avicularia dark brown and lophophore tentacles brown. Autozooids are rectangular, arranged in radiating linear series in the first layer, but mostly irregular in position and morphology in areas of frontal budding. Frontal shield granular, more so when the secondary calcification is extensive, with a marginal pore series. Primary orifice slightly wider than long, with the distal margin rounded and the proximal margin slightly bowed with a single rounded central sinus, very variable in size, and often pinched together at the top so that the sinus is almost closed, presenting considerable inter- and intra-colonial variability. Up to four widely spaced, distal oral spines are present in early ontogeny, with bases obscured by calcification, in most cases, only two are visible. The orifice rounded with a slight peristome, more raised proximally, with an umbo that supports an oval suboral-avicularium, perpendicular to the frontal plane and with a toothed distal rim. Vicarious avicularia large, spatulate, with dark brown spade-shaped columella and a parallel-sided rostrum. Ovicell noncleitral, widely open; covered by granular secondary calcification.

**Remarks**

*Celleporaria brunnea* was initially described in British Columbia by Hincks (1884), and the type specimen is preserved in the NHMUK (1886.3.6.33). It is a widespread species on the Pacific coast of North America, particularly in California (Soule 1961; Soule and Soule 1965; Soule et al. 1995; Winston 1986). Nevertheless, the native range is unclear, but the data suggest that the original distribution ranges from British Columbia to the Galapagos Islands, Ecuador (O'Donoghue and O'Donoghue 1926; Hastings 1929; Soule 1961; Soule and Soule 1965; Soule et al. 1995; Osman and Haugsness 1981; Keough and Downes 1982; Winston 1986). Recently, this species was recorded throughout the Mediterranean Sea with a swift expansion of its distribution (Koçak 2007; Çinar et al. 2008; Harmelin 2014 [as *Celleporaria* sp. aff. *brunnea*], Koçac and Aydin-Önen 2014; Lezzi et al. 2015; Lodola et al. 2015; Ferrario et al. 2016, 2018; Marić et al. 2017). It has also been recorded on the western Pacific coast from Korea (Seo and Min 2009; Lee et al. 2011) and more recently in Oahu, Hawai (McCuller and Carlton 2018). In 2013, *C. brunnea* was recorded for the first time in the Atlantic Ocean from a marina in Cascais, Portugal (Canning-Clode et al. 2013b), although this species has likely been present in the Atlantic at least since 2008 in the bay of Arcachon (André & Harmelin in DORIS 2021). Today, *Celleporaria brunnea* probably occurs in other areas of Portugal, particularly south of Lisbon because new records were detected in Arrabida and Algarve, mainly in harbours or on artificial substrates (see material examined section). Nevertheless, Vieira questioned the identity of some of these records (as personal communication in Harmelin 2014), noting certain differences between the material from Lebanon and specimens described from California. Harmelin (2014) described differences in the morphology of the small digitations from the sinus corners, and he considered the most evident differences to be in the sinus size and the size and frequency of interzooidal avicularia. Similar differences, such as in the sizes of the orifice and interzoidal avicularia or in sinus morphology, were also indicated by Lodola et al. (2015) and Lezzi et al. (2015) from Italian's specimens. Neither paperquestioned the identity of the species.

To solve this issue, the type specimen of *C. brunnea*, currently stored in the NHMUK (Fig. 12), was studied and compared with the Madeiran specimens. This comparison also included, data obtained from the figures in Winston (1986) and Soule et al. (1995) from Panama (Pacific coast) and Santa Barbara (California), respectively. Specimens from Lisbon and Algarve from the Atlantic coast as well as from Genova, Santa Margherita Ligure, La Spezia, Livorno, Olbia, Porto Torres, Porto Rotondo, Viareggio, Livorno and Caste Isardo, from the Mediterranean, were also included in the morphometric comparison. Furthermore, morphometric measurements of the orifice, length (including the sinus) and width, and size of the interzooidal avicularia were compared. A total of 384 measurements of orifices and 218 measurements of interzooidal avicularia were graphically compared (Fig. 13), and no significant differences were found between the colonies.

Here, we conclude that the morphological differences indicated by Harmelin (2014) apparently result from the high variability of certain characters. Thus, Harmelin indicated differences in the horizontal digitations in the sinus corners, which were observed to be very variable, as well as in the sinus size (Fig. 14). Other differences such as the frequency and size of the interzooidal avicularia, seem to be variable characters. The frequency was observed to be very variable from one part of the colony to another, and size seems to be related to the colony's ontogeny, being bigger in colonies with significant frontal development.

Hincks (1884) described only two lateral tall spines; nevertheless, in the type specimen studied, some zooids exhibit four oral spines, with the basal portion (the only preserved part) very variable in thickness (Fig. 12). This characteristic was also observed in specimens from Madeira with 4 or 2 oral spines on zooids of the colony margin to no spines in young zooids in other colony regions.

Considering the studied variability and the impossibility of sorting the specimens in different species based on morphological characters, the specimens from Madeira are considered to belong to *C. brunnea*. Records from Portugal by Canning-Clode et al. (2013b) also belong to this species. Moreover, the specimens studied, figured and described in the literature from the Mediterranean (Koçak 2007; Çinar et al. 2008; Koçac and Aydin-Önen 2014; Lezzi et al. 2015; Lodola et al. 2015, Ferrario et al. 2016, Marić et al. 2017), including the specimens recorded by Harmelin (2014, as *Celleporaria* sp. aff. *brunnea*), seem to belong to this species.

## Discussion and conclusion

The ten bryozoan species included in the current work are associated with fouling communities collected in the Madeira Archipelago on artificial substrates. Two of these species, *Crisia noronhai* sp. nov*.* and *Amathia madeirensis* sp. nov., are described as new to science. Although *C. noronhai* sp. nov*.* here is described as a new species, its presence in Madeira was previously recorded by Norman (1909) and identified as *C. eburnea*. In addition, no data were found in the literature that indicates the previous presence of the new species *Amathia madeirensis* sp. nov. on the island. Finally, *Crisia* sp. aff. *elongata* may also be considered a new species, but at this stage, a specific determination is not possible. A re-description of this species is necessary and should be addressed in future studies.

Notably, *B. ingens* was known only from its original description in Brazil (Vieira et al. 2012). This species adds to the list of macroinvertebrates originating from Brazilian and Caribbean waters and recently detected in Madeira (Wirtz and Canning-Clode 2009; Canning-Clode et al. 2013a, b; Ramalhosa et al. 2014; Souto et al. 2015, 2016). This also includes the bryozoans *Beania maxilladentata* Ramalho et al., 2008 and *Parasmitina alba* Ramalho et al., 2011. These two species, previously known from the Brazilian coast (Ramalho et al. 2008, 2011; Vieira et al. 2010), were also recently recorded in Madeira (Souto et al. 2015, 2016).

*Celleporaria brunnea* is recorded in Madeira for the first time, and new data and SEM figures for *T. inopinata* are also provided. Both species are well known as NIS along European coasts, where their distribution is spreading very rapidly along the Atlantic and Mediterranean coasts. Morphological comparison of specimens from Madeira and other European localities with the type specimen confirmed the identity of the Atlantic and Mediterranean material as *C. brunnea*.

*Scruparia ambigua* is recorded here for the first time in Madeira. Since this species typically grows on artificial substrates and is commonly found in harbours and marinas around the world (Ryland 1965; Beukhof et al. 2016; McCuller and Carlton 2018; Coolen et al. 2020), its presence in Madeira suggests a recent introduction via shipping. As this species forms small, repent, and erect uniserial colonies, in most cases growing on other organisms such as other bryozoans or hydroids, colonies could also have gone unnoticed in previous works.

Furthermore, *C. bertholleti*, *C. insularis*, and *S. maderensis*, previously recorded in Madeira Island from natural substrates (Norman 1909; Vieira et al. 2013; and Busk 1860; 1861), are now also found growing on artificial substrates (Canning-Clode et al. 2013a, b; Souto et al. 2015; Ramalhosa et al. 2019; this study).

With regard to the distribution of the species between the localities studied in the current work (Table 11), only one species, *Crisia noronhai* sp. nov., was recorded in both marinas and aquaculture facilities (except for the Porto Santo sampling site). Five of the species studied were recorded in the aquaculture facilities of Caniçal, three of them, *S. ambigua*, *C. insularis* and *T. inopinata*, were recorded only from here, and *C. bertholletii* was shared in Campanário, Calheta, and Porto Santo. *Bugula ingens* was recorded only from the offshore aquaculture farm of Campanário, and *S. maderensis* was recorded only from the recreational marina of Quinta do Lorde. *Crisia* cf. *elongata*, *Amathia madeirensis* sp. nov., and *Celleporaria brunnea* were collected only in recreational marinas.

**Table 11:** Distribution of the species according to anthropogenic activity and locality

	Aquaculture facilities		Recreational marinas			
	Campanário	Caniçal	Calheta	Funchal	Quinta do Lorde	Porto Santo
*Crisia noronhai* sp. nov.	x	x	x	x	x	
*Crisia* aff.* elongata*			x	x		
*Amathia madereirensis* sp. nov.			x			
*Scruparia ambigua*		x				
*Bugula ingens*	x					
*Cradoscrupocellaria bertholletii*	x	x	x			x
*Cradoscrupocellaria insularis*		x				
*Scrupocaberea maderensis*					x	
*Tricellaria inopinata*		x				
*Celleporaria brunnea*					x	x
Total species	3	5	4	2	3	2

Bryozoans are an important component in fouling communities, and this study highlights the importance of monitoring programmes in ports for the early detection of NIS. Furthermore, the use of PVC panels has proven to be quite effective in collecting bryozoans, and further studies should be promoted to disentangle the identity and distribution of bryozoans, which would help broaden our knowledge on bryozoan richness in this hotspot area.

## Declarations

### Sampling and field studies

All necessary permits for sampling and observational field studies have been obtained by the authors from the competent authorities and are mentioned in the acknowledgements.

### Data availability

All data generated or analyzed during this study are included in this published article.
